# Vaginal treatment of endometrial cancer: role in the elderly

**DOI:** 10.1186/1477-7819-9-74

**Published:** 2011-07-13

**Authors:** Massimo Moscarini, Enzo Ricciardi, Alessandro Quarto, Paolo Maniglio, Donatella Caserta

**Affiliations:** 1 Department of Women's Health and Territorial Medicine. Sapienza University of Rome. Sant'Andrea Hospital, Roma, Italy

**Keywords:** Endometrial Cancer, Surgery, Elderly Patients, Hysterectomy, Prognosis

## Abstract

**Background:**

To compare abdominal hysterectomy, the most currently used for treating cancer of the endometrium, to the vaginal hysterectomy in term of survival, morbidity and failure rates.

**Methods:**

We retrospectively analyzed 68 cases divided into two sub-groups. A study group of 31 cases received vaginal surgery; a control group of 37 cases was treated with a laparotomy. Mean operative time, median hospital stay, intra- and post-operative complications, DFS and OS time as well as occurrence of local or distant recurrences have been evaluated and reported. Cases included patients with a higher rate of medical morbidities (p = 0.01) than controls.

**Results:**

Mean age was 76.2 and 70.4 years in the vaginal (V) group and abdominal (A) group respectively. Mean operative time was longer for the group A. Group V patients had a lower mean post-operative hospital stay (p < 0.05). Differences in the two groups regarding intra- and post-operative complications, occurrence of local or distant recurrences and DFS time were not statistically significant. Disease specific survival time at 5 years scored 97% for group V, and 97% for group A.

**Conclusions:**

Results show how vaginal approach had a similar outcome in selected patients. Vaginal surgery could therefore be the proper choice in patients with early stages and lower surgical risk, in addition to elderly patients exposed to a higher surgical risk.

## Background

Endometrial carcinoma is the most common gynecological malignancy in western countries with an incidence of 15-20 per 100.000 women per year. In 2006, 41200 new cases were reported only in the United States with half of cases occurred in women older than 65 years [1].

Population aging is a major concern regarding this tumor. In 2030, 20% of the US population will be older than 65 [2]. This will increase the number of women affected by endometrial cancer, with a consistent raise of new cases per year. Among these new cases, elderly patients will play a major role in the statistics.

The current gold standard for endometrial cancer treatment is hysterectomy with BSO as well as peritoneal washing and pelvic and para-aortic lymphadenectomy, performed either thru a laparotomy (the majority of cases) or a laparoscopy. This is been performed according to FIGO revised surgical and pathologic staging [3,4].

Several prognostic factors have been identified. Tumor histology, stage and patient age seem to play an important role in survival [5].

Morbidities like cardiovascular disease, diabetes mellitus and obesity are frequent in the elderly. When they are concurrent to endometrial cancer, they raise surgical morbidity and mortality rates. Nevertheless, surgery is still mandatory for endometrial cancer staging and treatment [6].

According to literature, higher age at the time of surgery is associated to a worst prognosis. This evidence relates certainly to the fact that older patients have a higher chance to be under-treated, since their medical conditions do not allow a major surgery required to extirpate the tumor [7]. A less-invasive surgical approach appears to be the best choice among this group of patients. Nevertheless, it is mandatory to perform a procedure that assures equivalent cure rates.

Avoiding a major abdominal surgery and general anesthesia is highly remarkable. Less invasive approaches as laparoscopy have today shown an evidence-based equal treatment efficacy for early stages. Key-hole surgery allows a shorter recover and lower post-operative morbidity [8]. Nevertheless, these procedures could be often severely contra-indicated in endometrial cancer patient, since they require a general anesthesia, which is contra-indicated in endometrial cancer patients with frequent and concurrent morbidities. These results show how vaginal approach had a similar outcome in selected patients.

We compared the clinical outcome of the vaginal versus the abdominal hysterectomy in a population of elderly patients as treatment for endometrial cancer at an early stage. Morbidity, mortality, rates of recurrence, disease-free survival (DFS) and overall survival (OS) rates were evaluated and compared in both groups. The primary objective of the study was to evaluate the role of vaginal hysterectomy in elderly women with endometrial carcinoma.

## Materials and methods

We retrospectively reviewed a series of women older than 70 years who had a diagnosis of FIGO stage I or stage II endometrial endometrioid adenocarcinoma. These patients were consecutively treated at our center from April 2002 to June 2006. Unfavorable histologies were excluded from the series.

Two groups were identified. A first group (group V) included medically compromised women undergoing vaginal hysterectomy for cancer. A second group (group A) included patients who underwent abdominal surgery for cancer.

Group V considered patients with risk factors for surgery as hypertension (systolic pressure > 140 mmHg and/or diastolic pressure > 90 mmHg or patients treated with antihypertensive drugs), diabetes mellitus (basal glycemia > 140 mg/dL or patients treated with insulin or oral therapy), obesity (BMI > 30 kg/m2), massive obesity (BMI > 40 kg/m2), cardiovascular diseases (history of coronary artery disease (CAD), acute myocardial infarction, heart failure, transient ischemic attack (TIA) or stroke), respiratory diseases (obstructive or restrictive patterns). Controls had, on the other hand, an apparent good medical status.

Use of American Society of Anesthesiology (ASA) classes assessed surgical risk; cases were included in ASA class IV. Group V patients were considered unfit to general anesthesia at the anesthesiologist evaluation.

Patients were clinically staged by chest X-rays, abdomino-pelvic computed tomography (CT) scans or whole abdomen magnetic resonance imaging (MRI), and trans-vaginal ultrasound (US).

A pre-operative histological diagnosis of endometrial cancer was obtained in both groups on endometrial biopsy specimens.

Group V patients underwent a total vaginal hysterectomy with bilateral salpingo-oophorectomy at a time that included a vaginal margin being at least 1,5 and maximum 2 cm. Anesthesiologists always performed a spinal anesthesia.

Group A was treated with abdominal hysterectomy with the same vaginal margin extension as above, bilateral salpingo-oophorectomy, peritoneal washing and pelvic and para-aortic node dissection. A general anesthesia was performed in all group A cases.

All the cases (both groups) who showed a high grade (grade 3), deep myometrial invasion (> than a half) or a FIGO stage II at histology were addressed to adjuvant radiotherapy.

Mean operative time, mean hospital stay, intra- and post-operative complications, DFS and OS time and the occurrence of local or distant recurrences were then evaluated.

Follow-up protocol included: recto-vaginal examination, Pap smear from the vaginal cuff, total body CT scans every 6 months; chest X-rays and mammography on a yearly basis.

Mean follow-up was 45 months for group V (range 36-70), and 49 months for group A (range 36-72). A follow-up time of 36 months was considered valid according to literature's evidence that considers a higher risk of recurrence during the first 3 years that follow surgery[9].

Data are expressed as mean ± standard deviation. Non-parametric Mann-Whitney test, or χ2 test were used to compare data. Survival curves were plotted by means of Kaplan Meier method and compared by using the Log rank test. A p value lower than 0.05 was considered to be statistically significant

## Results

68 cases older than 70 years with a diagnosis of endometrial cancer were eligible for our study: 31 had vaginal surgery (group V); 37 underwent abdominal surgery (group A).

Vaginal surgery was performed in 45.6% (31/68) of patients, abdominal surgery in 54.4% (37/68). Group V patients' age range was 70-86 years, with a mean age of 76.2 years and a median of 74 years. Group A range was 66-84 years, with a mean age of 70.4 years and a median of 70 years.

Cases had a significant higher prevalence of co-morbidities (p = 0.01), obesity (p = 0.02) and cardiovascular disease (p = 0.04) (table 1).

**Table 1:** Data related to comorbidity in two groups of patients

	**Abdominal surgery(n = 37)%**	**Vaginal surgery(n = 31)%**	**p value**
	
CV disease	16.2(6)	51.6(16)	0.004
Hypertension	54.1(20)	74.2(23)	NS
DM	16.2(6)	25.8(8)	NS
Obesity	10.8(4)	51.6(16)	0.002
Other	48.6(18)	64.5(20)	NS

NS non significant: p > 0.05

56 patients (82%) presented at least one co-morbidity. 16 patients in the V group had three, or up to three risk factors. Only 3 patients showed a similar condition among controls.

All patients from group V had a spinal anesthesia. Mean operative time was 78 minutes (range 55-110) whether mean hospital stay was 6.6 days (range 5-10). Group A patients had all general anesthesia. Mean operative time was 131 minutes (range 115-200) whether mean hospital stay was 7.9 days (range 6-20) (table 2). 2 patients (2.9%) had intra-operative bleeding. One patient was from the V group, the other among group A.

**Table 2:** Data related to hospital stay and operative time in two groups of patients

	Abdominal surgery(n = 37)%	Vaginal surgery(n = 31)%	p value
Anesthesia			
general	100	-	< 0.005
spinal	-	100	< 0.005
Hospital stay (mean time)	9.1(± 2.6SD)	6.6((± 1.3SD)	< 0.005
Median operative time(min)	131 (115-200)	78(55-110)	< 0.005

6 patients (8.8%) experienced post-operative complications. In the V group, 2 patients developed a pelvic infection, in 1 patient a post-operative bleeding occurred. The A group counted 3 patients who had, respectively, bleeding, lymphorrea and deep venous thrombosis (table 3). No peri-operative deaths occurred.

**Table 3:** Intra and post operative complications in the two groups

	Abdominal surgery(n = 37)%	Vaginal surgery(n = 31)%	p value
Intraoperative complications			NS
Bleeding	1	1	
Postoperative complications			NS
Bleeding	1	1	
Lymphorrea	1	-	
Pelvic infection	-	2	
Deep Venous Thrombosis	1	-	

Distribution for stage, grade and myometrial depth invasion between groups is reported in table 4.

**Table 4:** Clinical and pathologic data relating to 68 patients undergoing vaginal or laparotomic surgery for endometrial cancer

	Abdominal surgery(n = 37)%	Vaginal surgery(n = 31)%	p value
Mean age(years)	70.4 (± 4.2SD)	76.2(± 5.6SD)	
FIGO stage			NS
IA	1(2.7%)	10(32.3%)	
IB	22(59.5%)	14 (45.2%)	
IC	11(29.7%)	6(19.4%)	
IIA	1(2.7%)	1(3.2%)	
IIB	2(5.4%)	-	
Histological Grade			NS
G1	4(10.8%)	8(25.8%)	
G2	18(48.6%)	21(67.7%)	
G3	15(40.5%)	2(6.5%)	
Myometrial depth invasion			0.03
M1(< 50%)	23(62.2%)	29(93.5%)	
M2(> 50%)	14(37.8%)	2(6.5%)	
Adjuvant RT			0.04
Yes	14(20.5%)	8(11.7%)	
No	23(33.8%)	23(33.8%)	

Patients submitted to adjuvant pelvic radiation therapy and vaginal brachytherapy were 6 (19%) with FIGO stage IC and 1 (3%) with FIGO stage II tumors, all from the V group.

All group A patients received pelvic and para-aortic node dissection. The mean number of pelvic/aortic nodes harvested was 11.5 ± 9.7 (1-34). No node metastases were found at histologic examination.

Among group A, 11 (30%) patients with FIGO stage IC tumors and 3 (8%) patients with stage II underwent adjuvant pelvic RT and vaginal brachytherapy.

During follow-up, 9 cases showed recurrences, which caused 2 patients to die of the disease. 2 (6%) patients from group V had local recurrence after 18 and 25 months, respectively, whether group A showed local recurrences after 26 and 58 months in 2 (5%) cases.

Distant recurrences occurred in 2 (6%) patients in vaginal surgery group after 12 and 35 months. Abdominal surgery group counted 3 (8%) cases after 6,12 and 18 months. 1 disease-related death (3%) and 3(9%) deaths from other causes occurred in the V group. Group A included 1(3%) death disease-related and 3(8%) deaths from other causes.

**Figure 1:**
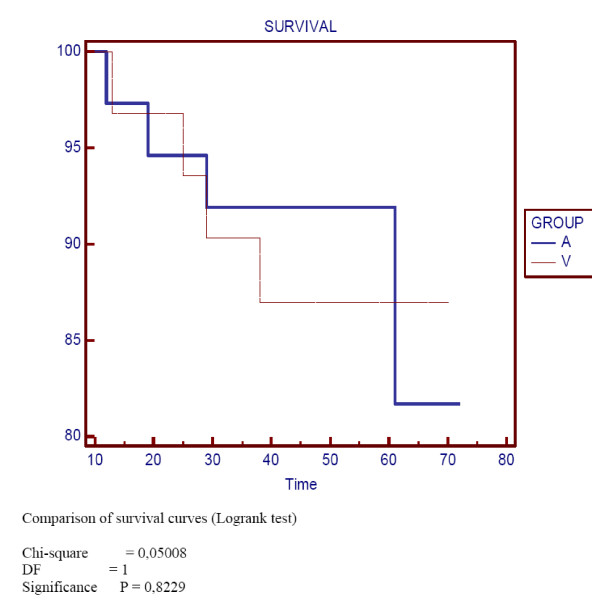
**Overall survival**.

**Figure 2:**
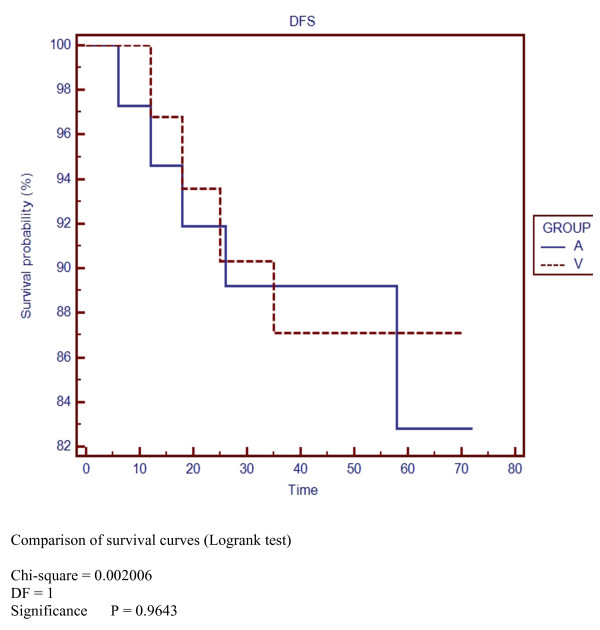
**Disease-free survival**.

**Figure 3:**
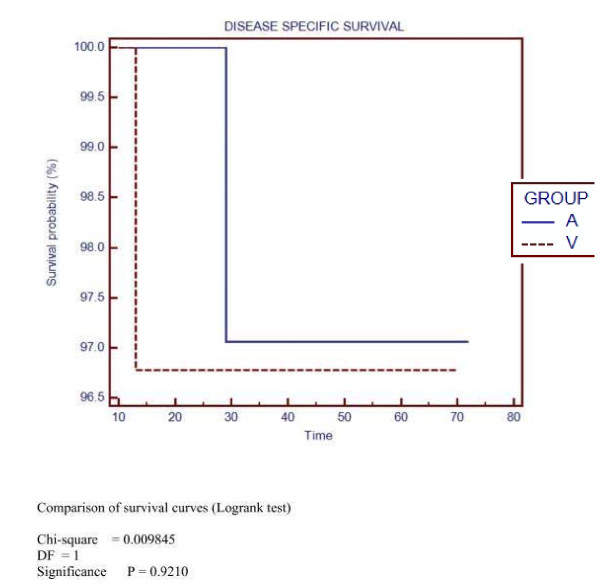
**Disease specific survival**.

5-years overall survival (OS) was 82% and 87% for group A and V respectively (NS) (Figure 1). Disease-free survival (DFS) at 5-years was 83% and 87% for group A and V respectively (NS) (Figure 2). Disease-specific survival at 5-years was 97% for both groups (NS) (Figure 3).

## Conclusions

Endometrial cancer accounts for the 7% of all women's cancer. Prognostic features are well defined. They include race, FIGO stage, tumor grade, depth of myometrial invasion, metastatic disease to pelvic and/or para-aortic nodes, cervical or adnexal involvement, histologic sub-types, presence of LVSI, DNA aneuploidy. FIGO stage is critical [4].

Current gold standard for both staging and treatment is surgery. It includes thorough exploration of the abdominal-pelvic cavity, pelvic washing, hysterectomy, bilateral salpingo-oophorectomy and pelvic and para-aortic lymphadenectomy. Alternative approaches include vaginal hysterectomy with vaginal bilateral salpingo-oophorectomy, first line radiation therapy and endocrine therapies [10,11]. Laparoscopic surgery is been progressively integrated into standard endometrial cancer care during the past years. Beside the well-known advantages, it is still unfit for patients who are poor candidates for general anesthesia. Age and obesity are relative contraindications. Difficulties in establishing pneumo-peritoneum and ventilation, poorer visualization, inability to tolerate Trendelenburg position are common problems encountered with obese patients. Laparoscopic surgery should be performed with an acceptable rate of complications to be a viable option, therefore it was not considered for group V women. Moreover, since data on long-term follow-up and recurrences are still unclear, it was preferred a comparison with standard abdominal procedure.

It is been cleared that a clinical, non surgical, approach has a very high risk of failure.

Elderly patients have a higher prevalence of comorbidities as obesity, diabetes mellitus, hypertension, CAD [12]. Thus, surgical risk for abdominal procedures is eventually higher among these patients and vaginal surgery appears safer. Nevertheless, it does not allow exploration of peritoneal contents. Therefore, an assessment of lymph nodal status is unachievable.

Obese women with high-level estrogens usually harbor a cancer diagnosed at an early stage, mostly IA or IB. Trimble et al. stated that lymphadenectomy in patients with a stage IA or IB do not provide a clear survival benefit [13]. 514 patients with early stage endometrial carcinoma were considered in a study to assess the role of systematic pelvic lymphadenectomy in improving survival rates. Surgical staging is statistically improved by this procedure while overall and disease-free survival is not different from patients who do not undergo a pelvic node dissection [14]. Recent prospective randomized trials as ASTEC claimed to unveil the nebulous scenario that surrounds lymph nodal dissection in endometrial cancer surgery [15].

The role of lymphadenectomy is still debated, since surgical staging procedures were incomplete and authors failed to assess the para-aortic area [16]. The recent revision of 1988 FIGO staging does not clarify whether a lymph nodal dissection should be performed or not. This is because a clear assessment of which patient should be considered low-risk or high-risk is still missing. Moreover, a standardization of lymph node dissection appears to be necessary. A standardized procedure should include a precise definition of the anatomic margins and specify the extent of dissection, as well as state clearly how many lymph nodes should be harvested.

In our study, we assessed survival rates in a population of elderly patients with early stage endometrial cancer, who presented a higher surgical risk. These patients underwent a vaginal hysterectomy in place of the standard abdominal procedure. Comparing this approach to the traditional procedure used in a control group, we got evidences of high-cure rate achieved in elderly patients with the vaginal technique (> 70 years old). This evidence has been confirmed in other series in literature [7,17].

A follow-up of at least 3 years showed that medium-term survival of both groups was similar.

Patients who underwent vaginal hysterectomy presented massive obesity (BMI > 40 kg/m2), hypertension and diabetes mellitus more frequently than other group patients (p < 0.005). Intra-operative complications were not statistically significantly different between the two groups. Controls had a higher frequency of post-operative complications, probably related to the more extensive procedure. Mean hospital stay and operative times were significantly lower for group V.

Results show how vaginal surgery associated or not to adjuvant radiation therapy is a feasible and valid approach in elderly patients with comorbidities and early-stage of the disease.

Vaginal surgery could therefore be the proper choice in selected patients with early stages and lower surgical risk [18], in addition to the elderly patient exposed to a higher surgical risk.

## Conflict of Interest Statement

The authors declare that they have no competing interests.

## Authors' contributions

MM participated in design of the study and revisions, gave intellectual input and corrected the manuscript. ER conceived of the study, and participated in its design and coordination, performed statistics and drafted the manuscript. AQ collected clinical data and performed statistics. PM participated in collecting data and read and corrected the manuscript. DC helped in editing, read and corrected the manuscript. All authors read and approved the final manuscript.
